# Mediastinitis in pediatric cardiac surgery: an unusual complication. A single-center experience

**DOI:** 10.47487/apcyccv.v6i2.471

**Published:** 2025-06-27

**Authors:** Laura Isabel Manosalva Arciniegas, José Antonio Vargas Soler, Lyneira Juliana Guevara Rodríguez, Laura Constanza González Hakspiel, Andrés Felipe Rubio Duarte, Sara Elena Mendoza Crespo

**Affiliations:** 1 Fundación Cardiovascular de Colombia, Floridablanca, Colombia. Fundación Cardiovascular de Colombia Floridablanca Colombia

**Keywords:** Mediastinitis, Pediatrics, Cardiovascular Surgery, Negative-Pressure Wound Therapy, Mediastinitis, Pediatría, Cirugía Cardiovascular, Terapia de Presión Negativa para Heridas

## Abstract

**Objectives.:**

Mediastinitis is an unusual postoperative complication in pediatric cardiovascular surgery, associated with high morbidity and mortality. Case reports are limited, especially in Latin America, where cardiac surgery has advanced significantly.

**Materials and methods.:**

A retrospective study of mediastinitis post-cardiac surgery cases at a high-complexity center in Colombia, between January 2015 and June 2023, was conducted. A detailed description of the clinical characteristics and therapeutic approaches was provided.

**Results.:**

A population of 16 children with mediastinitis was characterized, predominantly male (68.7%); 62.5% were aged 1-3 months. The most common defects were transposition of the great arteries and univentricular heart. Gram-negative bacteria (37.5%) were the most common isolates, followed by gram-positive bacteria (25%). Vacuum-assisted closure (VAC) was required in 43.7% of cases.

**Conclusion.:**

Advances in congenital heart disease management have led to complications such as mediastinitis, prompting the development of therapeutic strategies that would benefit from better characterization in high-complexity centers to improve outcomes.

## RESUMEN

**Objetivo.:**

La mediastinitis es una complicación posoperatoria inusual en cirugía cardiovascular pediátrica, asociada con alta morbilidad y mortalidad. Los reportes de casos son limitados, especialmente en Latinoamérica, donde la cirugía cardíaca ha avanzado significativamente.

**Materiales y métodos.:**

Estudio retrospectivo de casos de mediastinitis pediátrica poscirugía cardíaca en un centro de alta complejidad en Colombia, entre enero de 2015 y junio de 2023. Se realizó una descripción detallada de las características clínicas y las estrategias terapéuticas.

**Resultados.:**

Se caracterizó una población de 16 niños con mediastinitis, predominantemente varones (68,7%). El 62,5% tenía edades de 1 a 3 meses. Los defectos más comunes fueron la transposición de las grandes arterias y el corazón univentricular. Las bacterias gramnegativas (37,5%) fueron los aislamientos más comunes, seguidas de las grampositivas (25%). Se requirió cierre asistido por vacío (VAC) en el 43,7% de los casos.

**Conclusiones.:**

Los avances en el manejo de las cardiopatías congénitas han provocado complicaciones como la mediastinitis, lo que ha impulsado el desarrollo de estrategias terapéuticas que se beneficiarían de una mejor caracterización en centros de alta complejidad para mejorar los resultados.

## Introduction

Mediastinitis is a devastating postoperative complication in pediatric cardiac surgery, with a global incidence of 0.4% to 5% and mortality ranging from 14% to 47%. ^1^^,^^2^ The increase in these healthcare-associated infections is linked to advancements in congenital heart defect treatment, which expose patients to multiple risk factors related to both pediatric physiology and the hospital environment. ^3^

Pediatric-specific factors include immune immaturity, genetic anomalies (e.g., DiGeorge syndrome, asplenia, Down syndrome), nasal *Staphylococcus aureus* carriage, resistant pathogen colonization, parenteral nutrition, age under one year, low weight, congenital heart disease complexity, and prior antibiotic use. ^1^^,^^4^

Similarly, surgical factors include urgency, prolonged surgery, aortic clamping, deep hypothermia, bone wax, extensive electrocautery, prosthetic implants (e.g., valves, Contegra® conduit, ventricular assist devices), cardiac electrodes, reintervention, extracorporeal membrane oxigenation (ECMO), and delayed sternal closure due to hemodynamic instability. ^5^^,^^6^

Characterizing mediastinitis is essential to identify prevalent etiologies and contributing factors. The limited number of reports in Latin American literature hinders a clear epidemiological understanding, restricting the development of prevention strategies and increasing healthcare costs due to associated morbidity and mortality. ^1^^,^^7^

The aim of this report is to describe a series of cases of mediastinitis in a high-complexity institution in Colombia, detailing clinical characteristics and the therapeutic strategies employed.

## Materials and methods

### Study Population

A retrospective descriptive case series was conducted by reviewing the medical records of patients under 18 years old who underwent cardiac surgery and were diagnosed with mediastinitis, based on CDC criteria ^8^, between January 1, 2015, and June 30, 2023, at a tertiary referral center for congenital heart disease in northeastern Colombia. 

### Variables

Demographic and clinical variables were collected, including details of medical and surgical management.

### Statistical Analysis

For the analysis of qualitative variables, proportions represented in percentages were calculated, while quantitative variables were expressed in medians and interquartile ranges, considering their non-normal distribution after the application of the Shapiro-Wilk test. Jamovi® was used for data processing.

### Ethical Considerations

This report was approved by the institutional ethics committee. All patient data were anonymized to ensure confidentiality and in accordance with the Declaration of Helsinki and Resolution 8430 of 1993.

## Results

Between 2015 and 2023, approximately 250 palliative or corrective cardiovascular surgeries were performed annually on pediatric patients with congenital heart disease, with a mediastinitis prevalence of 0.71% (95% confidence interval [CI]: 0.42%-1.12%) during the study period. The annual distribution of mediastinitis cases was consistent, ranging from 1 to 3 cases per year.

Sixteen cases were reported during this period and formed our study population. Regarding demographic characteristics, most mediastinitis cases occurred in males (68.7%), with the highest proportion in infants aged 1 to 3 months (62.5%). Among cardiac conditions, transposition of the great arteries was the most frequent cause of postoperative mediastinitis (25%), followed by hypoplastic aortic arch (18.9%) (Table 1). Notably, 31.3% of children with mediastinitis had a history of previous sternotomy, and 62.5% required a pre-surgical hospital stay longer than 14 days.

**Table 1:** Sociodemographic and preoperative characteristics.

Preoperative variables	Total population n = 16 (%)
Sex	
Male	11 (68.7)
Female	5 (31.3)
Age groups	
< 1 month	2 (12.5)
>1 month - < 3 months	10 (62.5)
> 3 months	4 (25)
Weight (kg), (median [IQR])	3.8 [3.5-4.7]
Type of congenital heart disease	
D-TGA	4 (25)
Hypoplastic aortic arch + VSD	3 (18.9)
HLHS	2 (12.5)
Atrioventricular canal	2 (12.5)
Tricuspid atresia	2 (12.5)
Pulmonary atresia + VSD	1 (6.2)
Anomalous pulmonary venous drainage	1 (6.2)
Ventricular septal defect	1 (6.2)
Previous sternotomy	
Yes	5 (31.3)
No	11 (68.7)
Days of Pre-surgical Hospitalization (median [IQR])	19 [13-42]
< 14 days	6 (37.5)
>14 Days	10 (62.5)
Preoperative infections	
Yes	4 (25)
No	12 (75)

IQR: Interquartile range. D-TGA: Dextro-transposition of the great arteries. VSD: Ventricular septal defect. HLHS: Hypoplastic left heart syndrome.

Accordingly, 62.6% of the patients received antibiotic prophylaxis with vancomycin and amikacin for 24 hours, which was extended to 3 days in 12.5% of cases due to an open sternum. The antibiotic regimens were guided by the institutional protocol based on the bacterial susceptibility profile of the intensive care unit.

Prosthetic materials were used more frequently (56.2%) than autologous pericardium for congenital defect repair. Open sternum was required in 56.2% of patients during the postoperative period. While no children required ECMO in the preoperative period, 31.2% required it during the postoperative period.

**Figure 1:**
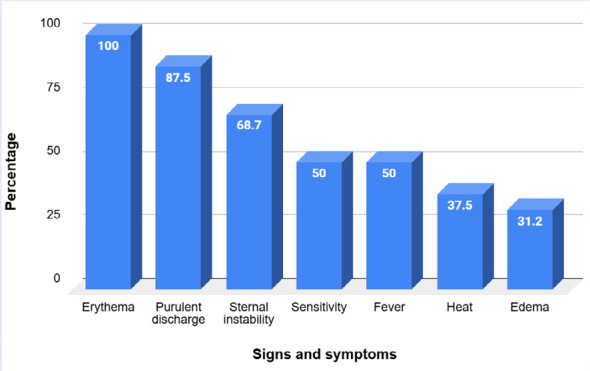
Signs and symptoms of mediastinitis in pediatric cardiac surgery.

In mediastinitis cases, the median time from surgery to the onset of symptoms was 14 days (IQR 11.5-17.5). The most common signs were erythema, purulent discharge, and sternal instability, followed by fever and skin tenderness. Abscesses and localized edema were also observed (Figure 1). Intraoperative findings during sternal lavage revealed bone involvement in 25% of cases.

Paraclinical tools at the onset of mediastinitis signs showed elevated white blood cell counts and C-reactive protein levels in most cases (Table 2). Gram stain of skin discharge was positive in only 37.5% of children.

**Table 2:** Intraoperative and postoperative variables.

Intraoperative and postoperative variables	Total population n = 16 (%)
Antibiotic prophylaxis	
Vancomycin - Amikacin	10 (62.6)
Cefazolin - Amikacin	4 (25)
Clindamycin - Amikacin	1 (6.2)
Vancomycin - Cefepime	1 (6.2)
Type of prosthetic and non-prosthetic material	
Goretex	5 (31.3)
Goretex + stent	1 (6.2)
Goretex + Bovine Pericardium	1 (6.2)
Autologous pericardium	5 (31.3)
Bovine pericardium	2 (12.5)
None	2 (12.5)
Total surgery time (min), (mean [SD])	288 [146]
Pump time (min), (mean ± SD)	166 ± 141
Clamp time (min), (mean ± SD)	68.7 ± 61.6
Postoperative open sternum	
Yes	9 (56.2)
No	7 (43.8)
Open sternum days	
Less than 3 days	5 (55.6)
More than 3 days	4 (44.4)
Postoperative ECMO	
Yes	5 (31.2)
No	11 (68.8)
Postoperative ICU days (median [IQR])	57.5 [37-79]
Mortality	
Yes	1 (6.2)
No	15 (93.8)
Day of onset of infection in relation to surgery (median [RIC])	14 [11.5-17.5]
Total leukocyte count u/L(mean ± SD)	17233 ± 8669
C-reactive protein (mg/L) (mean ± SD)	157 ± 127

IQR: Interquartile range. SD: Standard Deviation. u/L: units per liter. mg/L: milligrams per liter.

**Figure 2:**
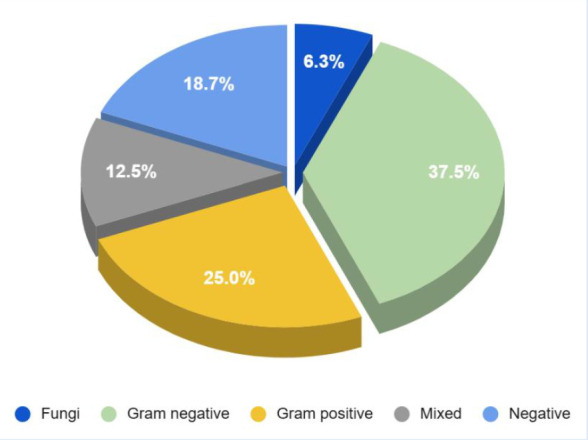
Microbiological profile of cultures in mediastinitis.

Of the microorganisms isolated from mediastinal secretion cultures, 37.5% were gram-negative, 25% were gram-positive, and 6.3% were fungi; no microorganisms were found in 18.7% of cultures. Mixed infections occurred in 2 cases (gram-positive with gram-negative bacteria and fungi with gram-negative bacteria) (Figure 2).

Broad-spectrum antibiotics were recommended for 4 weeks in most cases, except in 3 cases that did not require Vacuum-assisted closure (VAC) therapy, with 2 cultures yielding negative results. Antibiotics were prescribed for 6 weeks in only two cases of osteomyelitis and mediastinitis.

Mediastinal lavage was performed in all cases; however, in 43.7% of the children, a VAC system was installed, which required pressure between 50 mmHg and 75 mmHg, with no reports of hemodynamic compromise during its use, which in most cases was less than 2 weeks.

The group of children requiring VAC therapy was predominantly male infants, around one month old, and diagnosed with complex congenital heart disease. In the postoperative period, over 50% required an open sternum for at least three days. Microbiological isolates were primarily gram-negative bacteria, followed by mixed infections with Candida spp. and methicillin-sensitive Staphylococcus aureus.

VAC therapy typically lasted about 9 days; however, in one case, it was extended to 26 days due to a mediastinal abscess identified during surgery, requiring four sequential sponge changes every 4-6 days. Treatment included 4 weeks of broad-spectrum antibiotics such as vancomycin, carbapenems, cephalosporins, and occasionally caspofungin.

## Discussion

Advances in the treatment of complex congenital heart disease have increased the number of children undergoing palliative or corrective surgery. This presents physiological challenges, including surgical and anesthetic risks, and potential hematologic, pulmonary, renal, and neurological complications, creating a favorable context for severe postoperative complications such as mediastinitis. ^9^^-^^10^

Few reports exist on the epidemiological situation in the pediatric population, with an incidence of less than 5% and morbidity and mortality rates ranging from 12% to 50%. ^11^^)^ Although cardiac surgery is considered a clean procedure in a sterile environment, the incidence of mediastinitis exceeds expectations, with underreporting suggesting an uncertain epidemiological profile, especially in Latin America. ^12^^)^ In this retrospective study, we found a prevalence of 0.7%, significantly lower than the 3.3% in Guatemala and 3.5% in Chile. ^13^^,^^14^

Risk factors are primarily shared between adult and pediatric populations, though significant differences exist, particularly in genetic alterations, primary and secondary immunodeficiencies, severe malnutrition, and intraoperative and postoperative factors. ^14^

A factor such as age is a key factor influencing the likelihood of mediastinitis, often linked to the timing of surgical intervention. Most patients in case series are between 1 and 12 months old, with a male predominance. Similarly, in our population, 62.5% of children were aged 1 to 3 months, with a 2:1 male-to-female ratio. ^15^^,^^16^

The congenital heart diseases most associated with mediastinitis include univentricular physiology, tetralogy of Fallot, and interventricular septal defects. However, most reports do not specifically describe the direct relationship with any type of heart disease. ^16^

Regarding pathophysiological mechanisms, intraoperative contamination of the mediastinal cavity may occur due to host skin colonization by specific microorganisms, suboptimal preoperative disinfection, surgical team expertise, and even the quality of operating room air, which may introduce contaminated particles into the surgical field. ^1^

Pathogens involved in mediastinitis primarily originate from endogenous sources (patient’s microbiota), exogenous sources (surgical materials, operating room air), and the surgeon. The most frequent cause is endogenous microbiota, predominantly methicillin-sensitive *Staphylococcus aureus* and coagulase-negative *Staphylococci*, followed by Gram-negative bacteria such as *Klebsiella pneumoniae*, *Escherichia coli*, and *Pseudomonas spp.*^7^^,^^14^^,^^15^ This microbiological pattern contrasts with the present study, where 37.5% of isolates were Gram-negative bacteria, some of which were BLEE-positive and producers of metallo-β-lactamases. Gram-positive bacteria were isolated in 25% of cultures, followed by fungi at 6.3%.

Recently, the CDC published new criteria for diagnosing mediastinitis based on clinical and paraclinical findings, with specific guidelines for children under 1 year. Key criteria include microbiological isolation from mediastinal tissue or fluid, fever >38°C, pain, sternal instability, purulent drainage, or mediastinal widening. For infants under 1 year of age, hypothermia or bradycardia is also considered. Additional signs like erythema, pain on palpation, and wound dehiscence also support the diagnosis. ^8^ Additional diagnostic tools include elevated CRP, leukocytosis, positive mediastinal secretion cultures (gold standard), findings on invasive devices or electrodes, and imaging such as ultrasound. ^8^

For diagnosis, chest X-rays (AP and lateral) may show air along the sternum or sternal separation. Chest CT is the most commonly used preoperative tool, detecting fluid, gas, or sternal diastasis, though changes may appear up to 2 weeks after onset. Bone scintigraphy with labeled leukocytes can help diagnose sternal osteomyelitis but has technical limitations in children. ^15^^,^^17^ Additionally, ultrasound can reveal signs suggestive of local inflammation, such as retrosternal fluid accumulation and increased echogenicity of parasternal fat. When correlated with other imaging and clinical findings, its diagnostic performance for mediastinitis improves. ^18^

Mediastinitis management requires aggressive surgery and prolonged antibiotic therapy (4-6 weeks). Key steps include surgical revision with debridement, removal of infected material, collection of samples for culture and antibiotic sensitivity, and intravenous antibiotics adjusted to pathogen susceptibility. ^1^^,^^2^^,^^19^

In relation to surgical strategies, several techniques have been mentioned, each with its own set of advantages and disadvantages. These include delayed sternal closure, primary thoracic closure with continuous antibiotic irrigation, muscle flaps, and VAC. ^19^ However, the VAC system has become one of the therapeutic pillars for the management of mediastinitis, being a relatively recent technique first described by Argenta and Morykwas in 1997 and subsequently applied by Obdeijn in a case of post-sternotomy mediastinitis in an adult patient in 1999. ^20^^,^^21^ Since then, it has been used more frequently in children with favorable results, although there are few reports on its use, particularly in the Latin American literature. ^21^^)^ Sherman *et al*. reported one of the largest mediastinitis case series, involving 50 children who underwent cardiac surgery in Israel, treated with 5 days of VAC therapy (50-75 mmHg) and 4 weeks of antibiotics. The most common pathogen was methicillin-sensitive *Staphylococcus aureus*. ^16^

In our pediatric population, 43.7% required VAC management for 8 to 14 days, combined with antibiotic therapy targeting primarily gram-negative bacteria, with consideration of fungi and gram-positive bacteria. The criteria for initiating VAC therapy included persistent sternal instability despite initial surgical intervention, ongoing wound dehiscence with purulent drainage, or sustained elevation of white blood cell count and C-reactive protein despite antibiotic treatment, potentially associated with fever or septic shock, or radiological evidence on CT of changes consistent with mediastinitis and osteomyelitis. The remaining children who did not require VAC were managed with 1 to 2 sternal lavages and antibiotic therapy for 4 to 6 weeks, with strict follow-up of cultures from samples obtained during the lavages, which were negative, and monitoring of hemogram and CRP levels, which needed to decrease, along with the resolution of inflammatory signs at the wound site.

The VAC system applies negative pressure (25-100 mmHg) through a polyurethane sponge. This range is recommended to avoid adverse cardiac effects, such as reduced cardiac output, particularly in pediatric patients with congenital heart disease undergoing intracardiac or cardiopulmonary procedures, which could simulate a clinical scenario similar to cardiac tamponade. ^19^^)^ Advantages of this technique include hermetic sealing of the mediastinum to prevent contamination, improved drainage to reduce exudate and inflammatory cytokines, decreased tissue edema limiting bacterial colonization, prevention of residual cavities, facilitation of early sternal closure and thoracic stability, and enhanced microcirculation and granulation tissue formation, promoting secondary closure. ^22^^,^^23^ Additional benefits of the VAC system include sternal support, reducing paradoxical motion, improving respiratory mechanics, and aiding early extubation and pulmonary rehabilitation. ^22^^,^^24^

For VAC use, the polyurethane foam should extend at least 4 cm beyond the skin edge, acting as a pseudosternum for thoracic stability. The system is used for 6 to 21 days, based on inflammation reduction, C-reactive protein levels, and negative mediastinal cultures. A drainage guide tube is essential, and the sponge should be replaced every 2 to 3 days until optimal conditions for sternal closure are achieved. ^2^^,^^21^^,^^25^^,^^26^

Although our cases are similar to those reported worldwide, which could be a limitation, it is one of the few reports of mediastinitis in the pediatric population in the postoperative period following cardiovascular surgery in Latin America. Given the advancements in the treatment of complex congenital heart disease, continued progress in characterizing this healthcare-associated infection is essential.

In conclusion, in our experience, mediastinitis after pediatric cardiac surgery had a prevalence of 0.7%, which was more frequent in male patients, with a predominant etiology of Gram-negative bacteria.
